# 1-{(*E*)-[4-(4-Meth­oxy­phen­yl)butan-2-yl­idene]amino}-3-methyl­thio­urea

**DOI:** 10.1107/S160053681201611X

**Published:** 2012-04-21

**Authors:** Ming-Yueh Tan, Thahira Begum S. A. Ravoof, Mohamed Ibrahim Mohamed Tahir, Karen A. Crouse, Edward R. T. Tiekink

**Affiliations:** a Department of Chemistry, Universiti Putra Malaysia, 43400 Serdang, Malaysia; b Department of Chemistry, University of Malaya, 50603 Kuala Lumpur, Malaysia

## Abstract

Two independent mol­ecules comprise the asymmetric unit of the title compound, C_13_H_19_N_3_OS, which differ in the conformations of the residues linking the thio­urea and the terminal benzene ring, as manifested in the C_m_—C_m_—C_a_—C_a_ torsion angles [78.03 (16) and −93.64 (16)°, respectively; m = methyl­ene and a = aromatic]. The dihedral angles [84.40 (4) and 88.28 (5)°] formed between the thio­urea residue and the benzene ring indicate an almost orthogonal relationship. In each thio­urea residue, the N—H hydrogen atoms are *anti*, and the terminal N—H hydrogen atom forms an intra­molecular N—H⋯N hydrogen bond with the imine-N atom. In each case, the conformation about the imine C=N double bond [1.2812 (17) and 1.2801 (17) Å] is *E*. In the crystal, the mol­ecules are connected by N—H⋯S hydrogen bonds and these are connected into four mol­ecule aggregates *via* N—H⋯O hydrogen bonds, which are assembled into a two-dimensional array parallel to (011) *via* C—H⋯π and π–π inter­actions [ring centroid–centroid distance = 3.8344 (9) Å].

## Related literature

For background to chalcone thio­semicarbazides, see: Zhang *et al.* (2011 ▶). For background to hydrazinecarbodithio­ates, see: Khoo *et al.* (2005 ▶); Chan *et al.* (2008 ▶); Ravoof *et al.* (2010 ▶). For related syntheses, see: Tian *et al.* (1997 ▶); Tarafder *et al.* (2002 ▶).
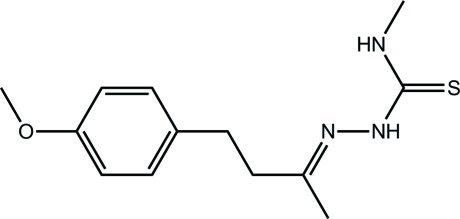


## Experimental

### 

#### Crystal data

C_13_H_19_N_3_OS
*M* *_r_* = 265.37
Triclinic, 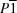

*a* = 9.6344 (4) Å
*b* = 11.1759 (6) Å
*c* = 13.4619 (8) Å
α = 80.324 (5)°
β = 87.103 (4)°
γ = 76.360 (4)°
*V* = 1388.48 (13) Å^3^
*Z* = 4
Cu *K*α radiation
μ = 2.01 mm^−1^
*T* = 100 K
0.41 × 0.23 × 0.14 mm

#### Data collection

Oxford Diffraction Xcaliber Eos Gemini diffractometer
Absorption correction: multi-scan (*CrysAlis PRO*; Agilent, 2011 ▶) *T*_min_ = 0.51, *T*_max_ = 0.75
18287 measured reflections
5312 independent reflections
4995 reflections with *I* > 2σ(*I*)
*R*_int_ = 0.022

#### Refinement

*R*[*F* ^2^ > 2σ(*F* ^2^)] = 0.037
*wR*(*F* ^2^) = 0.101
*S* = 1.01
5312 reflections
343 parameters
4 restraints
H atoms treated by a mixture of independent and constrained refinement
Δρ_max_ = 0.29 e Å^−3^
Δρ_min_ = −0.41 e Å^−3^

### 

Data collection: *CrysAlis PRO* (Agilent, 2011 ▶); cell refinement: *CrysAlis PRO*; data reduction: *CrysAlis PRO*; program(s) used to solve structure: *SHELXS97* (Sheldrick, 2008 ▶); program(s) used to refine structure: *SHELXL97* (Sheldrick, 2008 ▶); molecular graphics: *ORTEP-3* (Farrugia, 1997 ▶), *DIAMOND* (Brandenburg, 2006 ▶) and *Qmol* (Gans & Shalloway, 2001 ▶); software used to prepare material for publication: *publCIF* (Westrip, 2010 ▶).

## Supplementary Material

Crystal structure: contains datablock(s) global, I. DOI: 10.1107/S160053681201611X/qm2063sup1.cif

Structure factors: contains datablock(s) I. DOI: 10.1107/S160053681201611X/qm2063Isup2.hkl

Supplementary material file. DOI: 10.1107/S160053681201611X/qm2063Isup3.cml

Additional supplementary materials:  crystallographic information; 3D view; checkCIF report

## Figures and Tables

**Table 1:** Hydrogen-bond geometry (Å, °) *Cg*1 is the centroid of the C7–C12 ring.

*D*—H⋯*A*	*D*—H	H⋯*A*	*D*⋯*A*	*D*—H⋯*A*
N1—H1n⋯N3	0.87 (1)	2.15 (1)	2.5931 (16)	111 (1)
N4—H4n⋯N6	0.87 (1)	2.13 (1)	2.5818 (17)	112 (1)
N2—H2n⋯S2	0.87 (1)	2.70 (1)	3.5686 (11)	176 (1)
N5—H5n⋯S1	0.88 (1)	2.65 (1)	3.5276 (11)	178 (1)
N1—H1n⋯O2^i^	0.87 (1)	2.51 (2)	3.0979 (16)	125 (1)
C1—H1*B*⋯*Cg*1^ii^	0.98	2.94	3.5930 (17)	125

Symmetry codes: (i) 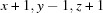; (ii) 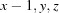.

**Fig. 1.**
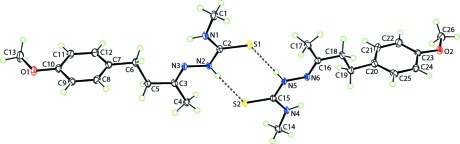
The molecular structure of the two independent molecules in (I) showing the atom-labelling scheme and displacement ellipsoids at the 50% probability level. Hydrogen bonds are shown as dashed lines.

**Fig. 2.**
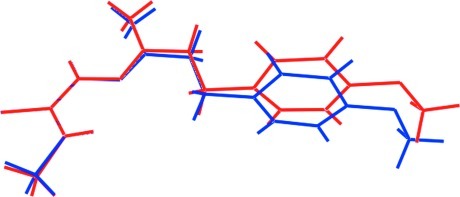
An overlay diagram of two independent molecules in (I). The S1-containing molecule is illustrated in red and the S2-molecule in blue. Molecules have been aligned so that the N1,S1,N2 and N4,S2,N5 planes are overlapped.

**Fig. 3.**
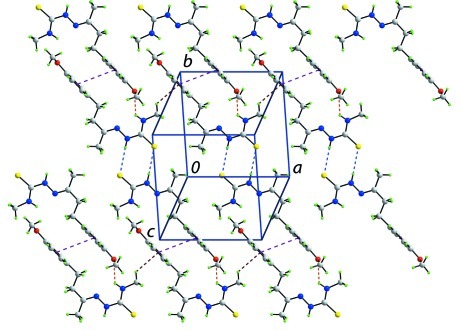
A view of the supramolecular layer parallel to (011) in (I) mediated by N—H···S, N—H···O, C—H···π and π–π interactions shown as blue, orange, brown and purple dashed lines, respectively.

**Fig. 4.**
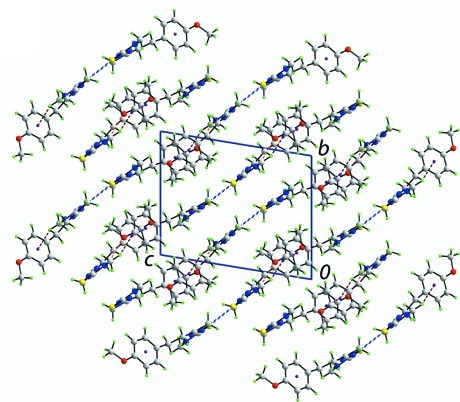
A view in projection down the a axis of the unit-cell contents for (I). The N—H···S, N—H···O, C—H···π and π–π interactions shown as blue, orange, brown and purple dashed lines, respectively.

## supplementary crystallographic information

### Comment

To initiate comparative studies between hydrazinecarbothioamide Schiff bases (Zhang *et al.*, 2011) and hydrazinecarbodithioate derivatives synthesized in our laboratory in on-going investigations (Khoo *et al.* 2005; Chan *et al.* 2008; Ravoof *et al.*, 2010), the title compound was synthesized and characterized crystallographically.

Two independent molecules comprise the asymmetric unit of (I), Fig. 1. As evidenced from the overlay diagram, Fig. 2, while the thiourea residues are super-imposable, minor conformational differences are seen in the links between these and the terminal benzene rings. Significant differences are noted between the chemically equivalent C5—C6—C7—C8 and C18—C19—C20—C21 torsion angles of 78.03 (16) and -93.64 (16)°, respectively. The dihedral angles formed between the thiourea residues (N1,C1,S1,N2 and N4,C15,S2,N5) and the benzene rings are 84.40 (4) and 88.28 (5)°, respectively. In each case, the methoxy group is co-planar with the benzene ring to which it is connected as seen in the values of the C13—O1—C10—C9 and C26—O2—C23—C22 torsion angles of 175.41 (12) and -5.62 (19)°, respectively. In each thiourea residue, the N—H hydrogen atoms are *anti*, and the terminal N—H hydrogen atom forms an intramolecular N—H···N hydrogen bond with the imine-N atom, Table 1. The conformation about the imine C═N double bond [N3═C3 = 1.2812 (17) Å and N6═C16 = 1.2801 (17) Å] is *E* in each case.

As indicated in Fig. 1, the independent molecules are connected by N—H···S hydrogen bonds leading to an eight-membered {···HNCS}_2_ synthon, Table 1. These are connected into four molecule aggregates *via* N—H···O hydrogen bonds, Table 1. The four molecule aggregates are assembled into a two-dimensional array parallel to (011) *via* C—H···π, Table 1, and π–π interactions occurring between the benzene rings [ring centroid(C7–C12)···centroid(C20–C25)^i^ distance = 3.8344 (9) Å with a tilt angle = 2.08 (7)° for symmetry operation *i*: 1 + *x*, -1 + *y*, 1 + *z*). Layers stack without significant intermolecular interactions between them, Fig. 4.

### Experimental

The title compound was synthesized following established literature procedures (Tian *et al.* 1997; Tarafder *et al.* 2002). To 4-methyl-3-thiosemicarbazide (1.05 g, 10 mmol) dissolved in hot absolute ethanol (25 ml) was added an equimolar amount of 4-(4-methoxyphenyl)butan-2-one (1.70 ml) also in hot absolute ethanol (20 ml). The mixture was stirred for about half an hour at ~340 K and then cooled to room temperature. The Schiff base precipitated was filtered and dried *in vacuo* over anhydrous silica gel. Colourless crystals were obtained after one week from a 1:1 mixture of 2-propanol and absolute ethanol. Yield 76%, *M*.pt. 356 K.

### Refinement

Carbon-bound H-atoms were placed in calculated positions (C—H = 0.95 to 0.99 Å) and were included in the refinement in the riding model approximation with *U*_iso_(H) set to 1.2 to 1.5*U*_equiv_(C). The amino H-atoms were refined with a distance restraint of N—H = 0.88±0.01 Å, and with *U*_iso_(H) = 1.2*U*_eq_(N).

### Crystal data

**Table:**

C_13_H_19_N_3_OS	*Z* = 4
*M**_r_* = 265.37	*F*(000) = 568
Triclinic, *P*1	*D*_x_ = 1.269 Mg m^−^^3^
Hall symbol: -P 1	Cu *K*α radiation, λ = 1.54180 Å
*a* = 9.6344 (4) Å	Cell parameters from 10673 reflections
*b* = 11.1759 (6) Å	θ = 3–71°
*c* = 13.4619 (8) Å	µ = 2.01 mm^−^^1^
α = 80.324 (5)°	*T* = 100 K
β = 87.103 (4)°	Prism, colourless
γ = 76.360 (4)°	0.41 × 0.23 × 0.14 mm
*V* = 1388.48 (13) Å^3^	

### Data collection

**Table:**

Oxford Diffraction Xcaliber Eos Gemini diffractometer	5312 independent reflections
Radiation source: sealed X-ray tube	4995 reflections with *I* > 2σ(*I*)
Graphite monochromator	*R*_int_ = 0.022
Detector resolution: 16.1952 pixels mm^-1^	θ_max_ = 71.6°, θ_min_ = 3.3°
ω/2θ scans	*h* = −11→11
Absorption correction: multi-scan (*CrysAlis PRO*; Agilent, 2011)	*k* = −13→13
*T*_min_ = 0.51, *T*_max_ = 0.75	*l* = −16→16
18287 measured reflections	

### Refinement

**Table:**

Refinement on *F*^2^	Primary atom site location: structure-invariant direct methods
Least-squares matrix: full	Secondary atom site location: difference Fourier map
*R*[*F*^2^ > 2σ(*F*^2^)] = 0.037	Hydrogen site location: inferred from neighbouring sites
*wR*(*F*^2^) = 0.101	H atoms treated by a mixture of independent and constrained refinement
*S* = 1.01	*w* = 1/[σ^2^(*F*_o_^2^) + (0.065*P*)^2^ + 0.5168*P*] where *P* = (*F*_o_^2^ + 2*F*_c_^2^)/3
5312 reflections	(Δ/σ)_max_ = 0.001
343 parameters	Δρ_max_ = 0.29 e Å^−^^3^
4 restraints	Δρ_min_ = −0.41 e Å^−^^3^

### Special details

**Table:**

Geometry. All s.u.'s (except the s.u. in the dihedral angle between two l.s. planes) are estimated using the full covariance matrix. The cell s.u.'s are taken into account individually in the estimation of s.u.'s in distances, angles and torsion angles; correlations between s.u.'s in cell parameters are only used when they are defined by crystal symmetry. An approximate (isotropic) treatment of cell s.u.'s is used for estimating s.u.'s involving l.s. planes.
Refinement. Refinement of *F*^2^ against ALL reflections. The weighted *R*-factor *wR* and goodness of fit *S* are based on *F*^2^, conventional *R*-factors *R* are based on *F*, with *F* set to zero for negative *F*^2^. The threshold expression of *F*^2^ > 2σ(*F*^2^) is used only for calculating *R*-factors(gt) *etc*. and is not relevant to the choice of reflections for refinement. *R*-factors based on *F*^2^ are statistically about twice as large as those based on *F*, and *R*- factors based on ALL data will be even larger.

### Fractional atomic coordinates and isotropic or equivalent isotropic displacement parameters (Å^2^)

**Table:**

	*x*	*y*	*z*	*U*_iso_*/*U*_eq_	
S1	0.20415 (3)	0.32321 (3)	0.48243 (2)	0.01907 (11)	
O1	1.27782 (10)	−0.24724 (9)	0.93804 (7)	0.0204 (2)	
N1	0.34458 (12)	0.15989 (11)	0.63195 (9)	0.0183 (2)	
H1N	0.4267 (12)	0.1245 (15)	0.6602 (12)	0.022*	
N2	0.47979 (11)	0.26896 (10)	0.52508 (8)	0.0144 (2)	
H2N	0.4854 (17)	0.3310 (12)	0.4776 (10)	0.017*	
N3	0.59623 (11)	0.19555 (10)	0.57947 (8)	0.0145 (2)	
C1	0.21511 (15)	0.11973 (14)	0.66758 (12)	0.0241 (3)	
H1A	0.1413	0.1919	0.6817	0.036*	
H1B	0.2356	0.0584	0.7293	0.036*	
H1C	0.1813	0.0818	0.6157	0.036*	
C2	0.34971 (14)	0.24605 (12)	0.55167 (10)	0.0147 (3)	
C3	0.71806 (14)	0.22229 (12)	0.56058 (10)	0.0147 (3)	
C4	0.74688 (14)	0.32895 (13)	0.48538 (10)	0.0182 (3)	
H4A	0.7270	0.3172	0.4174	0.027*	
H4B	0.8472	0.3322	0.4892	0.027*	
H4C	0.6853	0.4072	0.5003	0.027*	
C5	0.84347 (14)	0.13959 (13)	0.61947 (10)	0.0170 (3)	
H5A	0.8808	0.1903	0.6607	0.020*	
H5B	0.9199	0.1102	0.5713	0.020*	
C6	0.81204 (14)	0.02645 (13)	0.68873 (11)	0.0206 (3)	
H6A	0.7314	0.0545	0.7343	0.025*	
H6B	0.7824	−0.0286	0.6476	0.025*	
C7	0.93936 (14)	−0.04719 (13)	0.75113 (10)	0.0174 (3)	
C8	0.97580 (14)	−0.00550 (13)	0.83614 (10)	0.0174 (3)	
H8	0.9211	0.0709	0.8534	0.021*	
C9	1.08986 (14)	−0.07296 (13)	0.89598 (10)	0.0165 (3)	
H9	1.1128	−0.0427	0.9535	0.020*	
C10	1.17070 (14)	−0.18502 (13)	0.87166 (10)	0.0164 (3)	
C11	1.13875 (15)	−0.22751 (13)	0.78626 (11)	0.0206 (3)	
H11	1.1945	−0.3033	0.7685	0.025*	
C12	1.02383 (16)	−0.15758 (13)	0.72690 (11)	0.0209 (3)	
H12	1.0028	−0.1864	0.6682	0.025*	
C13	1.35572 (17)	−0.36669 (14)	0.91966 (12)	0.0280 (3)	
H13A	1.2909	−0.4232	0.9244	0.042*	
H13B	1.4311	−0.4003	0.9699	0.042*	
H13C	1.3988	−0.3588	0.8521	0.042*	
S2	0.50040 (3)	0.51322 (3)	0.32073 (2)	0.01570 (10)	
O2	−0.50895 (10)	0.90797 (9)	−0.24093 (7)	0.0197 (2)	
N4	0.35843 (12)	0.67030 (11)	0.16874 (9)	0.0175 (2)	
H4N	0.2787 (13)	0.6966 (15)	0.1361 (12)	0.021*	
N5	0.22689 (12)	0.55527 (10)	0.27342 (8)	0.0151 (2)	
H5N	0.2193 (17)	0.4984 (13)	0.3255 (9)	0.018*	
N6	0.11708 (12)	0.60815 (10)	0.20570 (8)	0.0156 (2)	
C14	0.48459 (14)	0.71634 (14)	0.13471 (11)	0.0214 (3)	
H14A	0.5147	0.7551	0.1875	0.032*	
H14B	0.4623	0.7782	0.0735	0.032*	
H14C	0.5618	0.6467	0.1202	0.032*	
C15	0.35546 (14)	0.58411 (12)	0.24972 (10)	0.0140 (3)	
C16	−0.00410 (14)	0.58042 (12)	0.22484 (10)	0.0152 (3)	
C17	−0.04030 (15)	0.49618 (14)	0.31617 (11)	0.0209 (3)	
H17A	0.0148	0.4107	0.3147	0.031*	
H17B	−0.1426	0.4982	0.3166	0.031*	
H17C	−0.0168	0.5244	0.3770	0.031*	
C18	−0.11934 (14)	0.63481 (13)	0.14771 (10)	0.0181 (3)	
H18A	−0.1999	0.6888	0.1793	0.022*	
H18B	−0.1550	0.5657	0.1287	0.022*	
C19	−0.07400 (15)	0.71084 (14)	0.05199 (11)	0.0225 (3)	
H19A	−0.0429	0.7827	0.0700	0.027*	
H19B	0.0088	0.6584	0.0212	0.027*	
C20	−0.19197 (14)	0.75844 (13)	−0.02456 (10)	0.0189 (3)	
C21	−0.20533 (16)	0.69260 (14)	−0.10058 (12)	0.0241 (3)	
H21	−0.1412	0.6142	−0.1027	0.029*	
C22	−0.31004 (16)	0.73782 (13)	−0.17421 (11)	0.0229 (3)	
H22	−0.3162	0.6911	−0.2260	0.027*	
C23	−0.40494 (14)	0.85161 (12)	−0.17094 (10)	0.0161 (3)	
C24	−0.39597 (14)	0.91792 (13)	−0.09373 (10)	0.0166 (3)	
H24	−0.4620	0.9952	−0.0905	0.020*	
C25	−0.29074 (14)	0.87139 (13)	−0.02154 (10)	0.0183 (3)	
H25	−0.2858	0.9173	0.0310	0.022*	
C26	−0.50890 (16)	0.84812 (14)	−0.32718 (11)	0.0236 (3)	
H26A	−0.4153	0.8393	−0.3609	0.035*	
H26B	−0.5828	0.8987	−0.3740	0.035*	
H26C	−0.5286	0.7655	−0.3056	0.035*	

### Atomic displacement parameters (Å^2^)

**Table:**

	*U*^11^	*U*^22^	*U*^33^	*U*^12^	*U*^13^	*U*^23^
S1	0.01121 (17)	0.02285 (19)	0.01953 (19)	−0.00212 (13)	−0.00427 (12)	0.00540 (14)
O1	0.0189 (5)	0.0203 (5)	0.0185 (5)	0.0022 (4)	−0.0064 (4)	−0.0012 (4)
N1	0.0115 (5)	0.0217 (6)	0.0190 (6)	−0.0038 (4)	−0.0029 (4)	0.0054 (5)
N2	0.0122 (5)	0.0158 (6)	0.0133 (5)	−0.0030 (4)	−0.0032 (4)	0.0036 (4)
N3	0.0124 (5)	0.0160 (5)	0.0134 (5)	−0.0012 (4)	−0.0033 (4)	0.0007 (4)
C1	0.0135 (6)	0.0271 (8)	0.0276 (8)	−0.0058 (6)	−0.0012 (6)	0.0089 (6)
C2	0.0122 (6)	0.0166 (6)	0.0147 (6)	−0.0021 (5)	−0.0018 (5)	−0.0020 (5)
C3	0.0131 (6)	0.0177 (6)	0.0130 (6)	−0.0036 (5)	−0.0012 (5)	−0.0010 (5)
C4	0.0138 (6)	0.0226 (7)	0.0164 (6)	−0.0055 (5)	−0.0020 (5)	0.0046 (5)
C5	0.0120 (6)	0.0200 (7)	0.0171 (6)	−0.0038 (5)	−0.0024 (5)	0.0032 (5)
C6	0.0152 (6)	0.0220 (7)	0.0224 (7)	−0.0057 (5)	−0.0056 (5)	0.0060 (6)
C7	0.0139 (6)	0.0188 (7)	0.0176 (6)	−0.0059 (5)	−0.0019 (5)	0.0056 (5)
C8	0.0151 (6)	0.0160 (6)	0.0187 (7)	−0.0024 (5)	0.0014 (5)	0.0019 (5)
C9	0.0165 (6)	0.0192 (7)	0.0136 (6)	−0.0055 (5)	−0.0002 (5)	0.0001 (5)
C10	0.0134 (6)	0.0197 (7)	0.0142 (6)	−0.0039 (5)	−0.0014 (5)	0.0031 (5)
C11	0.0221 (7)	0.0181 (7)	0.0192 (7)	−0.0007 (5)	−0.0024 (5)	−0.0017 (5)
C12	0.0253 (7)	0.0209 (7)	0.0166 (7)	−0.0060 (6)	−0.0059 (6)	−0.0003 (5)
C13	0.0268 (8)	0.0250 (8)	0.0263 (8)	0.0076 (6)	−0.0089 (6)	−0.0044 (6)
S2	0.01168 (16)	0.01806 (18)	0.01548 (17)	−0.00205 (12)	−0.00432 (12)	0.00184 (13)
O2	0.0198 (5)	0.0215 (5)	0.0161 (5)	0.0002 (4)	−0.0070 (4)	−0.0032 (4)
N4	0.0121 (5)	0.0198 (6)	0.0181 (6)	−0.0039 (4)	−0.0049 (4)	0.0057 (5)
N5	0.0121 (5)	0.0171 (6)	0.0139 (5)	−0.0029 (4)	−0.0038 (4)	0.0042 (4)
N6	0.0132 (5)	0.0169 (6)	0.0149 (5)	−0.0021 (4)	−0.0042 (4)	0.0014 (4)
C14	0.0158 (6)	0.0245 (7)	0.0231 (7)	−0.0090 (6)	−0.0034 (5)	0.0058 (6)
C15	0.0132 (6)	0.0136 (6)	0.0150 (6)	−0.0018 (5)	−0.0016 (5)	−0.0032 (5)
C16	0.0134 (6)	0.0162 (6)	0.0151 (6)	−0.0026 (5)	−0.0014 (5)	−0.0005 (5)
C17	0.0150 (6)	0.0270 (8)	0.0191 (7)	−0.0076 (6)	−0.0027 (5)	0.0051 (6)
C18	0.0138 (6)	0.0224 (7)	0.0176 (7)	−0.0067 (5)	−0.0030 (5)	0.0030 (5)
C19	0.0152 (6)	0.0299 (8)	0.0199 (7)	−0.0077 (6)	−0.0044 (5)	0.0077 (6)
C20	0.0147 (6)	0.0236 (7)	0.0165 (7)	−0.0073 (5)	−0.0010 (5)	0.0061 (5)
C21	0.0217 (7)	0.0197 (7)	0.0258 (7)	0.0027 (6)	−0.0046 (6)	0.0015 (6)
C22	0.0256 (7)	0.0211 (7)	0.0211 (7)	−0.0012 (6)	−0.0049 (6)	−0.0054 (6)
C23	0.0146 (6)	0.0181 (7)	0.0143 (6)	−0.0050 (5)	−0.0026 (5)	0.0031 (5)
C24	0.0153 (6)	0.0172 (7)	0.0157 (6)	−0.0027 (5)	0.0002 (5)	0.0005 (5)
C25	0.0186 (6)	0.0248 (7)	0.0129 (6)	−0.0095 (5)	−0.0007 (5)	−0.0009 (5)
C26	0.0269 (7)	0.0262 (8)	0.0181 (7)	−0.0043 (6)	−0.0084 (6)	−0.0045 (6)

### Geometric parameters (Å, º)

**Table:**

S1—C2	1.6943 (13)	S2—C15	1.6881 (13)
O1—C10	1.3759 (16)	O2—C23	1.3736 (16)
O1—C13	1.4236 (17)	O2—C26	1.4341 (16)
N1—C2	1.3284 (18)	N4—C15	1.3319 (18)
N1—C1	1.4556 (17)	N4—C14	1.4530 (17)
N1—H1N	0.871 (9)	N4—H4n	0.869 (9)
N2—C2	1.3563 (17)	N5—C15	1.3616 (17)
N2—N3	1.3837 (15)	N5—N6	1.3860 (15)
N2—H2N	0.870 (9)	N5—H5n	0.875 (9)
N3—C3	1.2812 (17)	N6—C16	1.2801 (17)
C1—H1A	0.9800	C14—H14A	0.9800
C1—H1B	0.9800	C14—H14B	0.9800
C1—H1C	0.9800	C14—H14C	0.9800
C3—C4	1.4983 (18)	C16—C17	1.4974 (19)
C3—C5	1.5054 (17)	C16—C18	1.5055 (17)
C4—H4A	0.9800	C17—H17A	0.9800
C4—H4B	0.9800	C17—H17B	0.9800
C4—H4C	0.9800	C17—H17C	0.9800
C5—C6	1.5217 (19)	C18—C19	1.5242 (19)
C5—H5A	0.9900	C18—H18A	0.9900
C5—H5B	0.9900	C18—H18B	0.9900
C6—C7	1.5110 (18)	C19—C20	1.5119 (18)
C6—H6A	0.9900	C19—H19A	0.9900
C6—H6B	0.9900	C19—H19B	0.9900
C7—C12	1.388 (2)	C20—C21	1.384 (2)
C7—C8	1.395 (2)	C20—C25	1.396 (2)
C8—C9	1.3855 (19)	C21—C22	1.396 (2)
C8—H8	0.9500	C21—H21	0.9500
C9—C10	1.3908 (19)	C22—C23	1.3863 (19)
C9—H9	0.9500	C22—H22	0.9500
C10—C11	1.3893 (19)	C23—C24	1.3911 (19)
C11—C12	1.3952 (19)	C24—C25	1.3870 (19)
C11—H11	0.9500	C24—H24	0.9500
C12—H12	0.9500	C25—H25	0.9500
C13—H13A	0.9800	C26—H26A	0.9800
C13—H13B	0.9800	C26—H26B	0.9800
C13—H13C	0.9800	C26—H26C	0.9800
			
C10—O1—C13	116.80 (11)	C23—O2—C26	116.28 (10)
C2—N1—C1	123.67 (11)	C15—N4—C14	123.68 (11)
C2—N1—H1N	114.8 (11)	C15—N4—H4N	114.8 (11)
C1—N1—H1N	121.4 (11)	C14—N4—H4N	121.5 (11)
C2—N2—N3	117.48 (11)	C15—N5—N6	117.04 (11)
C2—N2—H2N	119.0 (11)	C15—N5—H5N	119.8 (11)
N3—N2—H2N	123.5 (11)	N6—N5—H5N	122.7 (11)
C3—N3—N2	118.22 (11)	C16—N6—N5	118.43 (11)
N1—C1—H1A	109.5	N4—C14—H14A	109.5
N1—C1—H1B	109.5	N4—C14—H14B	109.5
H1A—C1—H1B	109.5	H14A—C14—H14B	109.5
N1—C1—H1C	109.5	N4—C14—H14C	109.5
H1A—C1—H1C	109.5	H14A—C14—H14C	109.5
H1B—C1—H1C	109.5	H14B—C14—H14C	109.5
N1—C2—N2	116.68 (11)	N4—C15—N5	116.10 (11)
N1—C2—S1	123.03 (10)	N4—C15—S2	123.13 (10)
N2—C2—S1	120.28 (10)	N5—C15—S2	120.77 (10)
N3—C3—C4	125.83 (12)	N6—C16—C17	125.70 (12)
N3—C3—C5	117.16 (11)	N6—C16—C18	117.21 (11)
C4—C3—C5	117.01 (11)	C17—C16—C18	117.07 (11)
C3—C4—H4A	109.5	C16—C17—H17A	109.5
C3—C4—H4B	109.5	C16—C17—H17B	109.5
H4A—C4—H4B	109.5	H17A—C17—H17B	109.5
C3—C4—H4C	109.5	C16—C17—H17C	109.5
H4A—C4—H4C	109.5	H17A—C17—H17C	109.5
H4B—C4—H4C	109.5	H17B—C17—H17C	109.5
C3—C5—C6	115.30 (11)	C16—C18—C19	115.25 (11)
C3—C5—H5A	108.4	C16—C18—H18A	108.5
C6—C5—H5A	108.4	C19—C18—H18A	108.5
C3—C5—H5B	108.4	C16—C18—H18B	108.5
C6—C5—H5B	108.4	C19—C18—H18B	108.5
H5A—C5—H5B	107.5	H18A—C18—H18B	107.5
C7—C6—C5	112.58 (11)	C20—C19—C18	112.97 (11)
C7—C6—H6A	109.1	C20—C19—H19A	109.0
C5—C6—H6A	109.1	C18—C19—H19A	109.0
C7—C6—H6B	109.1	C20—C19—H19B	109.0
C5—C6—H6B	109.1	C18—C19—H19B	109.0
H6A—C6—H6B	107.8	H19A—C19—H19B	107.8
C12—C7—C8	117.58 (12)	C21—C20—C25	117.58 (12)
C12—C7—C6	121.97 (12)	C21—C20—C19	121.45 (13)
C8—C7—C6	120.45 (12)	C25—C20—C19	120.97 (13)
C9—C8—C7	121.52 (12)	C20—C21—C22	122.09 (13)
C9—C8—H8	119.2	C20—C21—H21	119.0
C7—C8—H8	119.2	C22—C21—H21	119.0
C8—C9—C10	119.84 (12)	C23—C22—C21	119.25 (13)
C8—C9—H9	120.1	C23—C22—H22	120.4
C10—C9—H9	120.1	C21—C22—H22	120.4
O1—C10—C11	124.80 (12)	O2—C23—C22	124.70 (12)
O1—C10—C9	115.30 (12)	O2—C23—C24	115.59 (12)
C11—C10—C9	119.89 (12)	C22—C23—C24	119.69 (12)
C10—C11—C12	119.18 (13)	C25—C24—C23	120.07 (12)
C10—C11—H11	120.4	C25—C24—H24	120.0
C12—C11—H11	120.4	C23—C24—H24	120.0
C7—C12—C11	121.94 (13)	C24—C25—C20	121.28 (13)
C7—C12—H12	119.0	C24—C25—H25	119.4
C11—C12—H12	119.0	C20—C25—H25	119.4
O1—C13—H13A	109.5	O2—C26—H26A	109.5
O1—C13—H13B	109.5	O2—C26—H26B	109.5
H13A—C13—H13B	109.5	H26A—C26—H26B	109.5
O1—C13—H13C	109.5	O2—C26—H26C	109.5
H13A—C13—H13C	109.5	H26A—C26—H26C	109.5
H13B—C13—H13C	109.5	H26B—C26—H26C	109.5
			
C2—N2—N3—C3	−174.61 (11)	C15—N5—N6—C16	178.41 (11)
C1—N1—C2—N2	−176.85 (13)	C14—N4—C15—N5	177.38 (12)
C1—N1—C2—S1	1.81 (19)	C14—N4—C15—S2	−2.45 (19)
N3—N2—C2—N1	4.06 (17)	N6—N5—C15—N4	6.56 (17)
N3—N2—C2—S1	−174.64 (9)	N6—N5—C15—S2	−173.61 (9)
N2—N3—C3—C4	1.38 (19)	N5—N6—C16—C17	1.1 (2)
N2—N3—C3—C5	−178.93 (10)	N5—N6—C16—C18	−177.38 (11)
N3—C3—C5—C6	5.04 (18)	N6—C16—C18—C19	3.64 (18)
C4—C3—C5—C6	−175.24 (12)	C17—C16—C18—C19	−174.98 (12)
C3—C5—C6—C7	−175.80 (11)	C16—C18—C19—C20	177.51 (12)
C5—C6—C7—C12	−102.04 (15)	C18—C19—C20—C21	−93.64 (16)
C5—C6—C7—C8	78.03 (16)	C18—C19—C20—C25	87.00 (16)
C12—C7—C8—C9	−1.6 (2)	C25—C20—C21—C22	2.1 (2)
C6—C7—C8—C9	178.36 (12)	C19—C20—C21—C22	−177.31 (13)
C7—C8—C9—C10	−0.1 (2)	C20—C21—C22—C23	−0.7 (2)
C13—O1—C10—C11	−3.41 (19)	C26—O2—C23—C22	−5.62 (19)
C13—O1—C10—C9	175.41 (12)	C26—O2—C23—C24	172.95 (12)
C8—C9—C10—O1	−177.48 (11)	C21—C22—C23—O2	177.50 (13)
C8—C9—C10—C11	1.4 (2)	C21—C22—C23—C24	−1.0 (2)
O1—C10—C11—C12	177.71 (12)	O2—C23—C24—C25	−177.41 (11)
C9—C10—C11—C12	−1.1 (2)	C22—C23—C24—C25	1.2 (2)
C8—C7—C12—C11	1.9 (2)	C23—C24—C25—C20	0.2 (2)
C6—C7—C12—C11	−178.01 (13)	C21—C20—C25—C24	−1.8 (2)
C10—C11—C12—C7	−0.6 (2)	C19—C20—C25—C24	177.55 (12)

### Hydrogen-bond geometry (Å, º)

Cg1 is the centroid of the C7–C12 ring.

**Table:**

*D*—H···*A*	*D*—H	H···*A*	*D*···*A*	*D*—H···*A*
N1—H1n···N3	0.87 (1)	2.15 (1)	2.5931 (16)	111 (1)
N4—H4n···N6	0.87 (1)	2.13 (1)	2.5818 (17)	112 (1)
N2—H2n···S2	0.87 (1)	2.70 (1)	3.5686 (11)	176 (1)
N5—H5n···S1	0.88 (1)	2.65 (1)	3.5276 (11)	178 (1)
N1—H1n···O2^i^	0.87 (1)	2.51 (2)	3.0979 (16)	125 (1)
C1—H1*B*···*Cg*1^ii^	0.98	2.94	3.5930 (17)	125

Symmetry codes: (i) *x*+1, *y*−1, *z*+1; (ii) *x*−1, *y*, *z*.
